# Supplementary material for: Predictors of ambulance transport to first health facility among injured patients in southern Sri Lanka
Source: PLoS One. 2021 Jun 25;16(6):e0253410. doi: 10.1371/journal.pone.0253410 (PMC8232418; doi:10.1371/journal.pone.0253410)
Supplement: S2 File — This is the English version of the survey that was used to collect the data for this analysis. (DOCX) [file pone.0253410.s002.docx]

Date (of data collection): ______/________/________

Day/month/year

02 / Apr /2017

Data Collector’s Initials: _____________

**Section A. Patient Background**

1. **Sex: €** Male **€** Female
2. **Age:** _______________ (years)
3. **Patient date of birth: ___________________________**

(day/month/year)

1. **Education: What is the highest level of school you have completed?**

**€** never attended school

**€** primary (Grades 1-5)

**€** junior secondary (Grades 6-9)

**€** vocational/technical training

**€** senior secondary (Grades 10-11)

**€** senior secondary GCE AL (Grades 12-13)

**€** university undergraduate

**€** postgraduate

**€** other (please specify): ____________________________________

1. **What is your occupation or job? ______________________________________**

**Relationship and Family**

1. **What is your current relationship status?**

**€** single

**€** married

**€** divorced

**€** widowed

**€** other (please specify):__________________________

1. **How many people live in the house or home where you reside?**

|  | **Adults (≥18 yrs)** |
| --- | --- |
|  | **Children** |

1. **What is your monthly household income (in rupees)? _____________________________**
2. **Does your household own a private vehicle?**

**€** no

**€** yes

**9a.** If yes, please specify type: **€** Bicycle

**€** 2-wheel motorized vehicle

**€** Tuk-Tuk, 3-wheel motorized

**€** 4-wheel motorized vehicle (car)

1. **Do you have any form of health insurance beyond what is provided through the universal coverage provided by the government?**

**€** no

**€** yes

1. **Do you have any significant chronic diseases?**

**€** no

**€** yes

**11 a.** If yes, please specify the condition(s):___________________________________________

**Section B. Injury Event**

**Now let’s think about the injury event.**

1. **What date did the injury occur? ______________________________________**
2. **What day of the week did the injury occur?**

**€** Monday **€** Friday

**€** Tuesday **€** Saturday

**€** Wednesday **€** Sunday

**€** Thursday

1. **About what time of day did the injury occur?** ______________________ am/pm

| 1. **Where did the injury occur? Please name the location and include the address.** |
| --- |
|  |

1. **What type of location were you (the patient) in when the injury occurred?**

**€** home/residence **€** market **€** sea/lake/river

**€** work **€** school **€** industrial/construction area

**€** street/highway **€** restaurant/bar

**€** farm **€** open land **€** other (specify): ______________________

1. **What was the mechanism or cause of injury?**

**€** traffic injury*** **(FILL OUT SECTION B1 TITLED “TRAFFIC INJURY ONLY”)**

**€** gun shot

**€** sexual assault **€** drowning **€** unknown

**€** fall **€** fire/heat **€** other (specify): _______________

**€** other blunt force **€** electricity

**€** stab or cut **€** choking/hanging

1. **Was this injury intentional or unintentional (Please check which is applicable)?**

**€** unintentional

**€** self-harm

**€** intentional (assault)

**€** unknown

**€** other (please specify): ______________________________

1. **Did you use alcohol within 6 hours of the injury event?**

**€** no

**€** yes

**€** prefer not to answer

***IF IT WAS A TRAFFIC INJURY PLEASE FINISH THIS SECTION (Section B) AND THEN FILL OUT SECTION B1. TRAFFIC INJURY**

**Section B1. TRAFFIC INJURY ONLY**

1. **How were you travelling when you were injured?**

**€** pedestrian **€** car

**€** non-motorized vehicle (bicycle) **€** pickup, van, minibus

**€** tuk-tuk **€** truck

**€** motorcycle **€** bus

**€** train **€** unknown

**€** other (specify): __________________________

1. **Type of Road User: What was the injured person (you) doing?**

**€** pedestrian

**€** driver or operator of the vehicle

**€** passenger

**€** unknown

**€** other (specify): ___________________________

**Section C. Prehospital Transportation and Care**

**Now let’s think about what happened before you got to the hospital. I will be asking questions about time of transportation, mode of transportation, and whether you received medical care on the way to the hospital.**

1. **Did you come to a health facility or hospital within an hour following injury?**

**€** no

**€** yes

**22a.** If no, how long did you wait before you decided to find a way to get to the hospital?

________ hours _________min

1. **Once you decided to seek care, did you have to wait for someone or transportation to take you to the health facility or hospital?**

**€** no

**€** yes

1. **If you did wait for transportation to the hospital, How long did you have to wait for transportation to the hospital?**

_____hours _____min

1. **Was there more than 1 part/leg on your trip to the hospital?**

**€** no

**€** yes

1. **Please fill out the table below regarding your trip to the hospital.**

Mode of Transport: walking, bicycle, personal car, tuk-tuk, police vehicle, carried, bus, taxi, private ambulance, train, motorcycle, other (specify)

| **Stage of Journey (from site of injury)** | **Mode of Transport** | **From (site of injury/dispensary/hospital name & district)** | **To (dispensary/hospital name & district)** | **Travel Time (list hours(hrs) and then minutes(min))** |
| --- | --- | --- | --- | --- |
| **1** |  |  |  |  |
| **2** |  |  |  |  |
| **3** |  |  |  |  |
| **4** |  |  |  |  |

1. **Did you receive treatment prior to coming to Karapitiya hospital?**

**€** no

**€** yes

| **27a.** If yes, what was done? |
| --- |
|  |

1. **Who administered this treatment before you got to the hospital?**

**€** trained nurse **€** doctor

**€** friend **€** family member

**€** trained lay responder **€** unknown

**€** bystander **€** other:_____________________________

1. **Where was this treatment administered?**

**€** at site of injury **€** district general hospital

**€** while travelling to hospital **€** private health facility

**€** non-specialist hospital **€** other (specify): ______________________________

**€** base hospital

1. **Did anybody travel with you to the hospital?**

**€** no

**€** yes

**30a.** If yes, what is their relation to you (specify)? _______________________________

1. **Did you (the patient) pay for transport to hospital?**

**€** no

**€** yes

**31a.** If yes, how much did you pay? ___________________________________________________

**Section D. Data Collection Form (TO BE OBTAINED FROM MEDICAL RECORD)**

**Now let’s get some information about the extent of the patient’s injury.**

1. **With regards to the injury type, please check all that apply to the patient seeking care/participant in our study.**

**Trauma Penetrating Trauma Blunt:**

**€** hip joint **€** hip joint

**€** thigh **€** thigh

**€** knee joint **€** knee joint

**€** lower leg **€** lower leg

**€** ankle **€** ankle

**€** foot **€** foot

**€** shoulder joint **€** shoulder joint

**€** upper arm **€** upper arm

**€** elbow **€** elbow

**€** forearm **€** forearm

**€** wrist **€** wrist

**€** hand **€** hand

**€** chest/abdomen/pelvis **€** chest/abdomen/pelvis

**€** head/neck/face **€** head/neck/face

**€** spinal cord/spine **€** spinal cord/spine

**Nature of Injury**

**€** fracture **€** concussion

**€** organ system injury **€** other traumatic brain injury

**€** open wound **€** bleeding

**€** burn **€** laceration

**€** cut **€** abrasion

**€** dislocation **€** aversion

**€** other (specify):_______________________________________

1. **Was there more than 1 body part affected?**

**€** no

**€** yes

| **33a.** If yes, indicate how many and specify parts affected |
| --- |
|  |

1. **Please indicate with an “X” all body parts that are affected by an injury.**

**
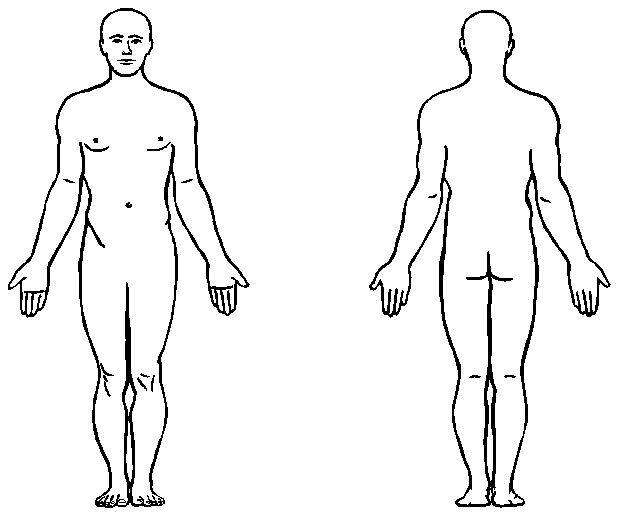
**

| **35. Please describe other important information about the patient’s injury severity. (ex. Amount of blood loss, type of fracture, whether patient was on ventilator or other life support, internal bleeding etc.)** |
| --- |
|  |

**Information on Initial Treatment Provided a Hospital-Treatment provided in ETC (Questions 36-44a.)**

1. **Was cardiopulmonary resuscitation (CPR) performed on patient upon arrival at hospital?**

**€** no

**€** yes

1. **Did patient require supplemental oxygen upon arrival or during initial treatment at hospital?**

**€** no

**€** yes

1. **Did patient require any hydration (IV Fluids) during initial treatment?**

**€** no

**€** yes

1. **Was patient given antibiotics?**

**€** no

**€** yes

| **39a.** If yes, please specify dose and brand/name: |
| --- |
|  |

1. **Was the patient given painkillers?**

**€** no

**€** yes

| **40a.** If yes, please specify dose and brand/name: |
| --- |
|  |

1. **Was the patient given other medications (not including antibiotics or painkillers)?**

**€** no

**€** yes

| **41a.** If yes, please specify dose and brand/name: |
| --- |
|  |

1. **Did the patient receive a blood transfusion?**

**€** no

**€** yes

| **42a.** If yes, please specify dose and brand/name: |
| --- |
|  |

1. **Did the patient have medical imaging?**

**€** no

**€** yes

**43a.** If yes, please specify type: **€** x-ray

**€** computed tomography (CT)

**€** magnetic resonance imaging (MRI)

**€** ultrasound

1. **Did the patient undergo any surgeries?**

**€** no

**€** yes

| **44a.** If yes, please describe type of surgeries |
| --- |
|  |

**Information About Treatment while in Hospital (EXCLUDING INITIAL/ETC TREATMENT)-Questions 45-53a.**

1. **Was cardiopulmonary resuscitation (CPR) performed on patient at any point since initial treatment?**

**€** no

**€** yes

1. **Did patient require supplemental oxygen any point past initial treatment?**

**€** no

**€** yes

1. **Did patient require any hydration (IV Fluids) during hospital stay (excluding initial treatment)?**

**€** no

**€** yes

1. **Was patient given antibiotics?**

**€** no

**€** yes

| **48a.** If yes, please specify dose and brand/name: |
| --- |
|  |

1. **Was the patient given painkillers?**

**€** no

**€** yes

| **49a.** If yes, please specify dose and brand/name: |
| --- |
|  |

1. **Was the patient given other medications (not including antibiotics or painkillers)?**

**€** no

**€** yes

| **50a.** If yes, please specify dose and brand/name: |
| --- |
|  |

1. **Did the patient receive a blood transfusion?**

**€** no

**€** yes

| **51a.** If yes, please specify dose and brand/name: |
| --- |
|  |

1. **Did the patient have medical imaging?**

**€** no

**€** yes

**52a.** If yes, please specify type: **€** x-ray

**€** computed tomography (CT)

**€** magnetic resonance imaging (MRI)

**€** ultrasound

1. **Did the patient undergo any surgeries?**

**€** no

**€** yes

| **53a.** If yes, please specify number and type of surgeries |
| --- |
|  |

**Mortality Information (to be filled by the data collector, not asked to the patient)**

1. **Did the patient die while in the hospital?**

**€** no

**€** yes

1. **If patient died while in hospital, what was cause of death?**

**___________________________________________________________________________________________________________**

1. **Where did patient spend time while in the hospital? Please check all that apply.**

**€** Operating room

**€** Surgical ward

**€** Intensive Care Unit

**€** Short stay ward

**€** Other (please specify): ___________________________________

1. **Date of admission**: _______________________________________
2. **Triage Score: 1 2 3 4**
3. **Date of discharge or death**: _____________________________
4. **If patient did not die while in hospital, what type of location was patient discharged to?**

**€** home

**€** another health facility

**€** other (please specify): ___________________________________________________________

1. **How many days was patient in the hospital before discharge or death (include day of admission as day 1)?**

________________ # of days
