# Supplementary material for: Predictors of ambulance transport to first health facility among injured patients in southern Sri Lanka
Source: PLoS One. 2021 Jun 25;16(6):e0253410. doi: 10.1371/journal.pone.0253410 (PMC8232418; doi:10.1371/journal.pone.0253410)
Supplement: Attachment — Submitted filename: PONE-D-21-02599_PLOS_REVIEW.docx [file pone.0253410.s003.docx]

Manuscript Number: **PONE-D-21-02599**

Article Type: Research Article

Full Title: **Predictors of ambulance transport to first health facility among injured patients in**

**Southern Sri Lanka**

Corresponding Author: Lindy M. Reynolds

**PEER REVIEW:**

The aim of the research to identify the proportion of injured patients who have been admitted to a tertiary hospital and who used an ambulance transportation to get to the first health facility have been well collected and the gained data was than examined on patient demographics, injury event, and injury type as predictors of ambulance transportation.

All scientific standards and procedures were adhered to and the procedure used for information gathering as well as the tools used for data analysis were presented.

The patient nbrs (n=405) do not represent an overwhelming data set but this has to be seen in the limitation described by the authors (relatively new EMS system; first research in this area; etc.). For figure 1, only 400 locations were provided and the authors missed to at least mention the missing injury locations of 5 patients.

The cross-sectional study nevertheless used descriptive statistics and tabulated the gained prehospital transportation variables in a reader friendly way.

The results of the study that 20.5% of patients used ambulance transportation to get to the first health facility in undoubtable a valuable source for information for prehospital emergency care providers in LMIC but does not represent scientifically unknow or surprising new knowledge. The factors that the researchers associated with ambulance use are of value for comparisons as these are the most often used indicators in the western world. Unfortunately do these westernized indicators (as well as most western researchers) often neglect the context the subject of research Emergency Medical Service (EMS) is imbedded in, and the obstacles that LMIC often present when it comes prehospital service implementation.

Some examples of obstacles:

- Is the ambulance service known by the affected patients (families/ bystanders/ population)?
- Is the EMS accessible and known (does a unified emergency nbr exist; is it really free or are hidden charges existing….)
- Are there laws and regulations existing (right of way for ambulances (traffic jams); lights & sirens use) and therefore is the really the fastest means transport (or are tuk-tuks much faster)?
- Is there any recognition (scope of practise/license) of care takers existing and to they really care (stabilize) for patients during transportation? (or may it be forbidden by law?)
- In case of RTI do emergency services like fire & police activate/ call EMS?

Some of these highly important sources of information the researchers looked into, others where limited due to the fact that the research was a hospital-based cross-sectional study. The other limitations of the study were explained well and the most limiting ones (patients younger than 18years and severely injured patients) could have probably been extrapolated or data could have been gathered later or from care takers (parents). Patients who rejected participating in the study are unfortunately not mentioned.

The conclusion that among lower acuity injury patients in southern Sri Lanka, 20.5% travelled in an ambulance to the first health facility, while others used other means of transport (over 50% used tuk tuks) is devastation for a young and prospering ambulance service but can serve well for further research needed to find out about the reasons behind. The predictors used by the researcher provide good information but may not represent the main barriers of EMS use.

**Comments to the Author:**

Things to be considered before publishing:

- The paper does not provide information about nbrs of patients who did not want to participate
- The missing (n=405 vs n=400) 5 patients from whom a location could not be determined should just be mentioned for completeness

**Comments to the Publisher:**

This is a short, informative piece that suits the online PLOS ONE journal well.

This paper examines the use of prehospital emergency care in Sri Lanka (SL), a country to which EMS is new and still in its infancy. The presents data that should be alarming for those responsible for implementing EMS in SL as part of countries Health Care Strengthening. It is a short research report based on a hospital-based cross-sectional study that does not provide or unveil new scientific evidence but it provides a good insider and new, highly valuable data. The used methodology is following all scientific standards and explained well.

As already elaborated, the study itself and its results may not have a huge scientific impact but it may raise awareness on political and planning level and may therefore be quite useful as well as it could stimulate further research on this highly important issue of pre-hospital emergency services.

It addresses all limitations and weaknesses. The discussion is short and the outcome may be limited due to as small sample size (n=405).

In short, I recommend this paper for publication in PLOS ONE online journal.

18. April 2021
